# Identification of the *Populus euphratica XTHs* Gene Family and the Response of *PeXTH7* to Abiotic Stress

**DOI:** 10.3390/plants14243847

**Published:** 2025-12-17

**Authors:** Jing Li, Hongyan Jin, Tongrui Song, Donghui Miao, Qi Ning, Jianhao Sun, Zhijun Li, Peipei Jiao, Zhihua Wu

**Affiliations:** 1 Xinjiang Production and Construction Corps Key Laboratory of Protection and Utilization of Biological Resources in Tarim Basin, College of Life Science & Technology, Tarim University, Alar 843300, China; jing926819729@163.com (J.L.); j2601803054@163.com (H.J.); songtr20001209@163.com (T.S.); mdh1216g@163.com (D.M.); ningqi20000423@163.com (Q.N.); sunjianhaotea@163.com (J.S.); lizhijun0202@126.com (Z.L.); 2 College of Life Sciences, Zhejiang Normal University, Jinhua 321004, China

**Keywords:** *Populus euphratica* Oliv., xyloglucan endotransglucosylase/hydrolase, gene family identification, stress response, growth and development

## Abstract

*Populus euphratica* Oliv. serves as a keystone species in desert riparian ecosystems. Owing to its pronounced tolerance to drought and salinity, as well as its robust reproductive capacity, it has become a pioneer species in desert oases. The xyloglucan endotransglucosylase (XET)/hydrolase (*XTH*) gene family plays a critical role in the remodeling of plant cell walls; however, its potential biological functions in poplar remain poorly understood. In this study, we identified the *XTH* gene family in *P. euphratica* and conducted a preliminary functional analysis. A total of 33 *PeXTH* genes were identified, which were unevenly distributed across the chromosomes, with the highest density observed on chromosome 6. Conserved domain analysis indicated that most members contain the typical GH16 domain associated with xyloglucan endotransglucosylase activity. Phylogenetic analysis classified them into four distinct subgroups, exhibiting evolutionary conservation with the model dicot plant of *Arabidopsis thaliana*. Notably, the promoter analysis revealed an abundance of ABA-responsive and stress-related cis-elements, suggesting their potential involvement in response to multiple stresses. Under drought stress, *PeXTH7* (*PeuTF07G00088.1*) exhibited a distinct expression pattern, with transcript levels significantly increasing with persistent treatment. RT-qPCR results confirmed that *PeXTH7* is highly expressed in both roots and leaves. Furthermore, subcellular localization assays demonstrated that the PeXTH7 protein localizes to the secretory pathway and the cell wall, implying a role in cell wall dynamic remodeling through the regulation of xyloglucan metabolism. The *PeXTH7*-overexpressing transgenic lines exhibited a significant increase in root length compared to the wild-type controls. As the first systematic analysis of the *XTH* gene family in *P. euphratica*, this study fills an important knowledge gap and provides new insights into the adaptive mechanisms of desert tree species.

## 1. Introduction

Under the pressure of intensifying global climate change and worsening water scarcity, drought stress has emerged as a major environmental constraint on agricultural productivity [1]. In response, plants have evolved sophisticated adaptive mechanisms that allow them to perceive external stress signals and mount appropriate physiological and molecular responses [2]. Studies have shown that the cell wall acts as the primary interface for plant–environment interactions, detecting external changes and relaying stress signals to the plasma membrane. This signaling cascade leads to changes in cytosolic Ca^2+^, reactive oxygen species (ROS), and hormone levels—including abscisic acid (ABA)—and further modulates the synthesis and remodeling of cell wall components through regulation of related gene expression, thereby enhancing plant adaptation to abiotic stress [3]. As the first barrier between plant cells and the external environment, the cell wall plays essential roles not only in sensing and transducing abiotic stress signals and maintaining structural integrity, but also in supporting plant growth and development [4]. The primary cell wall consists mainly of cellulose microfibrils, hemicellulose, pectin, and glycoproteins, which form a complex hydrated network. Water deficit can alter the composition of the cell wall, affecting turgor pressure regulation and growth dynamics, thereby modulating plant growth. Under stress conditions, plants upregulate genes involved in cell wall polysaccharide synthesis and modification through mechanisms such as stress-induced Ca^2+^ signaling oscillations and ABA-dependent pathways, thus preserving cell wall integrity and improving stress adaptability [5].

Xyloglucan (XG) serves as the predominant hemicellulose in the primary cell walls of dicotyledons and non-graminaceous monocotyledons. Its backbone consists of a β-(1 → 4)-linked D-glucan chain, which interacts with cellulose microfibrils via hydrogen bonds to form a complex network crucial for maintaining cell wall mechanical strength. Research has shown that under various abiotic stress conditions, transcription of genes involved in the lignin biosynthesis pathway is significantly upregulated. This transcriptional activation is accompanied by enhanced lignin deposition, which reinforces cell wall rigidity—an adaptive response that contributes to improved plant stress tolerance [6].

Xyloglucan endotransglycosylase/hydrolase (XTH), a key enzyme belonging to the glycoside hydrolase family 16 (GH16), catalyzes the cleavage and re-ligation of xyloglucan chains within the cellulose framework and is widely recognized as a pivotal regulator of cell wall remodeling. Through its modulation of xyloglucan metabolism, XTH dynamically regulates cell wall loosening and reinforcement, thereby directly influencing cellular elongation and expansion. Substantial evidence has demonstrated that plant roots exhibit greater sensitivity to abiotic stresses compared to other organs [7]. Functioning as the primary site for environmental stress perception, root growth displays a strong correlation with XTH-mediated xyloglucan metabolic dynamics, which may account for their enhanced stress responsiveness. Experimental findings indicate that when xyloglucan content in the Golgi apparatus substantially exceeds XTH levels, XTH incorporates into the cell wall primarily as xyloglucan-XTH complexes, resulting in enhanced wall rigidity and suppressed cell elongation. Conversely, under conditions where XTH abundance surpasses xyloglucan availability, XTH enters the cell wall as free enzymes that facilitate xyloglucan hydrolysis and transglycosylation, leading to wall loosening and promoted cell elongation [8].

*Populus euphratica* is a forest-forming tree species native to arid desert regions, primarily distributed across saline-alkali wastelands, desert areas, and river alluvial plains in northwestern China. As a pioneer species in desert riparian ecosystems, its remarkable adaptability to arid and saline-alkali environments establishes *P. euphratica* as a key woody model for studying plant responses to abiotic stresses such as drought and salinity [9,10]. Studies have demonstrated that under drought stress, *P. euphratica* seedlings exhibit thickening of the taproot, increased lateral root density, and significant modifications in root system architecture; these morphological adjustments contribute to enhanced water uptake and reduced water loss [11,12]. Furthermore, the dynamic remodeling of root system architecture (RSA) enables plants to directionally grow toward favorable soil conditions while avoiding adverse environments, thereby significantly improving their survival capacity [13].

Preliminary studies suggest that XTH primarily localizes to and functions in the primary cell wall, where it participates in regulating root cap development and the expression of genes related to root cap formation. By modulating cell wall elasticity and extensibility, XTH plays a significant role in plant adaptation to adverse environmental conditions [14]. Currently, 33, 29, 41, and 71 members of the *XTH* gene family members have been identified in *A.thaliana* [15], *Oryza sativa* [16], *Populus tomentosa* [17], and *wheat* [18], respectively. Increased expression of XTH strengthens the connection between primary and secondary walls in mesophyll cells by reducing the length of xyloglucan, which helps plants adapt to high temperatures [19]. In addition, *XTH* genes are also involved in regulating plant growth and development. Five *XTH* genes (*OfXTH24*, *27*, *32*, *35*, and *36*) in *Osmanthus fragrans* respond to changes in environmental temperature and participate in the regulation of flowering [20]. Transcriptomic analysis of *AcXTHs* in *Ananas comosus* indicates that it involved in the regulation of fruit ripening and Crassulacean acid metabolism (CAM) and exhibit tissue specificity. Real-time fluorescent quantitative PCR analysis shows that *AcXTH18* is involved in root growth of *A. comosus* [21]. Research has found that the *SsXTH* gene in *Schima superba* participates in glycoside metabolism through the transfer and hydrolysis of xyloglucan in the cell wall, thereby regulating the elongation of plant fibers [22]. During tomato fruit development, a novel *XTH* gene (*SlXTH5*) was identified with high expression in mature fruits, correlating with xyloglucan depolymerization [23]. Heterologous overexpression of the pepper *CaXTH3* in *A*. *thaliana* induced severe leaf curling, implying its role in cell wall remodeling and reinforcement, which may help protect mesophyll cells under drought stress [24]. In sugar beet, XTH has been shown to undergo changes during abiotic stress responses and is involved in stomatal development and function by modulating the flexibility and integrity of guard cell walls [25]. These findings collectively provide a foundation for understanding plant stress regulation mechanisms mediated by cell wall signaling. In recent years, with the release of high-quality genomes and multi-omics data from *Populus* plants, it has become easier to identify and analyze the biological function of *XTH* gene family [26,27,28].

In summary, *XTH* genes are closely associated with multiple aspects of plant growth and development, including root elongation, leaf venation patterning, fruit maturation, and stomatal development. Accumulating evidence demonstrates that XTH plays a crucial role in plant responses to abiotic stress, offering important insights into cell wall signal-mediated stress regulatory pathways. However, the potential function of the XTH family in the desert tree species *P. euphratica* remains elusive. In order to fill this knowledge gap, we identified, analyzed and predicted the *PeXTH* family, and functionally characterized the drought-responsive member *PeXTH7* to elucidate its role in root development and stress adaptation. Therefore, this study aims to identify and characterize the *XTH* gene family in poplar through genomic and bioinformatic analysis, with the objective of elucidating its potential functions in drought resistance. These results provide new insights for further understanding the role of *PeXTH* in response to abiotic stress, and also have important significance for the development and utilization of desert plant germplasm resources.

## 2. Results

### 2.1. Identification and Prediction of Physical and Chemical Properties of PeXTHs Family Members

A total of 33 *PeXTH* family members were identified in *P. euphratica*. The physicochemical properties of the encoded PeXTH proteins—including molecular weight (MW), amino acid number, instability index, theoretical isoelectric point (pI), grand average of hydropathicity (GRAVY), and subcellular localization—were analyzed using TBtools (version 2.119) and Expasy (https://web.expasy.org/protparam/; accessed on 24 November 2024). The results revealed that the molecular weights of PeXTH proteins range from 23.83 kDa to 39.49 kDa, with corresponding amino acid lengths varying between 210 and 338 residues. All members exhibit instability indices ranging from 55.81 to 71.41, indicating that they were generally unstable under in vitro conditions. The theoretical pI values vary widely from 4.48 to 9.58, likely reflecting differences in amino acid composition and polarity. Moreover, all GRAVY values were negative, confirming the overall hydrophilic nature of these proteins. Subcellular localization predictions suggest that 21 PeXTH proteins were localized to the cell wall, whereas the remaining 12 were predicted in both the cell wall and cytoplasm, implying potential roles in coordinating cell wall dynamics with intracellular signaling. Detailed physicochemical characteristics are provided in Table 1.

### 2.2. Analysis of Gene and Protein Structure of PeXTHs

**Figure 1:**
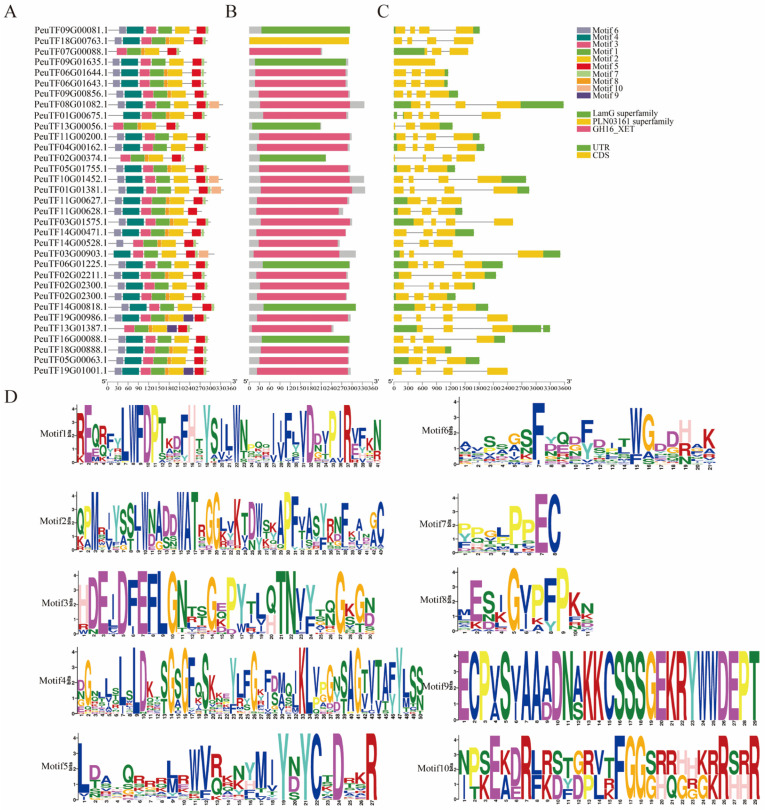
Analysis of gene structure and conserved structural domains of *PeXTHs*. (**A**) Distribution of conserved domains in *PeXTH*. (**B**) Pefam model of *PeXTH*; green indicates LamG superfamily, yellow indicates PLN03161 superfamily, and red indicates GH16_XET. (**C**) Gene structure of *PeXTH*. Yellow boxes indicate exons, black lines indicate introns, and green indicates non-coding regions. (**D**) Ten conserved motifs of PeXTHs, with single-letter abbreviations representing amino acids.

The gene distribution of conserved domains in *PeXTHs* and gene exon and intron group map were completed using the GSDS (Gene Structure Display Server) online platform. The prediction and visualization of conserved protein motifs were achieved with the help of the MEME (Multiple Em for Motif Elicitation) website. Bioinformatics analysis showed that the amino acid sequences of the *P. euphratica XTH* gene family contained 10 different characteristic motifs, among which motifs 1–7 and motif 10 were highly conserved in most members, while motif 9 was only present in three genes and showed low conservation (Figure 1A). Conservative domain analysis further confirmed that this gene family contains three different characteristic functional domains, among which GH16-XET (Glycoside Hydrolase Family 16 Xyloglucan Endotransglucosylase) is the main pfam model. This result is consistent with the reported catalytic domain characteristics of xyloglucan endotransglucosylase/hydrogenase (XTH) (Figure 1B). Gene structure analysis revealed that the number of exons in each member does not exceed four (Figure 1C); this minimalistic exon-intron arrangement pattern may be related to their functional conservation. The identification of conserved protein domains of *PeXTHs* revealed the presence of ten conserved motifs (motifs 1–10; Figure 1D). These findings suggest that the core functional regions are evolutionarily conserved.

### 2.3. Prediction of Cis-Acting Elements in PeXTHs Promoter Region

**Figure 2:**
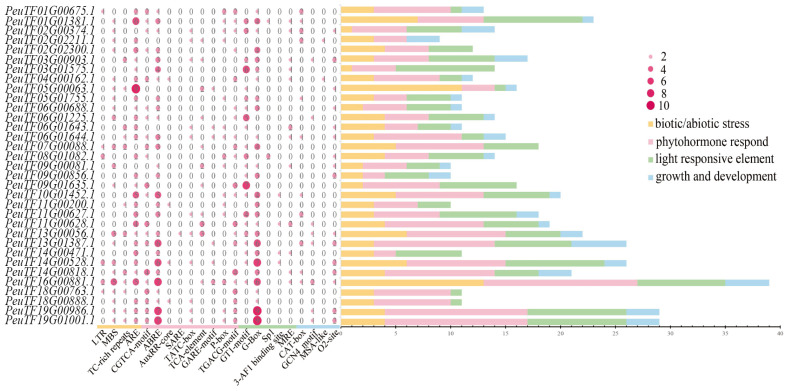
Statistics of cis-elements in the *PeXTHs* promoter region. The number in the figure represents the number of corresponding cis-acting elements predicted in the gene. The color depth and size of the circle are proportional to the number of components. The uncolored circle (number 0) indicates that the component is not detected. The color bars indicate the classification of cis-elements: yellow represents cis-elements related to biotic/abiotic stress, red represents cis-elements related to hormones, green represents cis-elements related to light response, and blue represents cis-elements related to growth and development.

The 2 kb promoter sequences upstream of the transcription start site (TSS) of all candidate genes were extracted, and cis-acting elements within these promoter regions were identified using online prediction tools. Systematic analysis revealed a total of 22 types of cis-regulatory elements, which were functionally categorized into four groups: abiotic/biotic stress-related, hormone-responsive, light-responsive, and growth/development-related elements. Among these, hormone-responsive elements were the most abundant, with nine types identified, including recognition sites for abscisic acid (ABA), methyl jasmonate (MeJA), gibberellin (GA), and salicylic acid (SA). ABA-responsive elements constituted the largest proportion within this category. Among the stress-related elements, hypoxia-responsive elements were predominant (Figure 2). These findings suggest that *PeXTH* genes may play diverse and important roles in regulating various physiological processes in *P. euphratica*, particularly under abiotic and biotic stress conditions. The presence of these cis-elements implies that the *PeXTH* gene family may be involved in modulating gene expression and multiple signaling pathways to enhance environmental stress adaptation.

### 2.4. Collinearity Analysis of XTHs Within P. euphratica and Among Multiple Species

**Figure 3:**
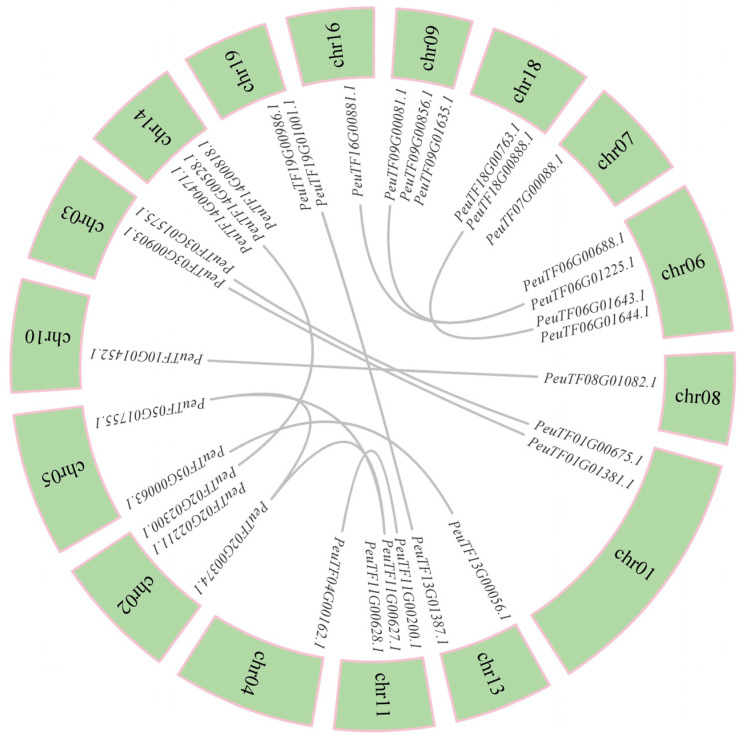
Intraspecific synteny analysis of 33 *PeXTHs* in *P. euphratica*. Green lines represent *P. euphratica* chromosomes, and gray lines indicate syntenic pairs of *PeXTH* fragment repeat genes.

By mapping the collinearity relationships among the 33 member genes within the *P. euphratica* gene family, 13 pairs of collinear *PeXTHs* genes were identified, including *PeuTF05G01755.1*, *PeuTF02G00374.1*, and *PeuTF11G00627.1*, each having two pairs of homologous genes, indicating that during the evolutionary process of *P. euphratica*, *XTHs* underwent multiple segmental duplications or repetitions, which facilitated the evolution and expansion of the gene family (Figure 3). Based on the collinearity relationships, 13 gene pairs were selected for selective pressure analysis, as shown in Table 2, with Ka/Ks values all less than 1, indicating that this gene family has been subjected to strong purifying selection pressure during evolution.

**Figure 4:**
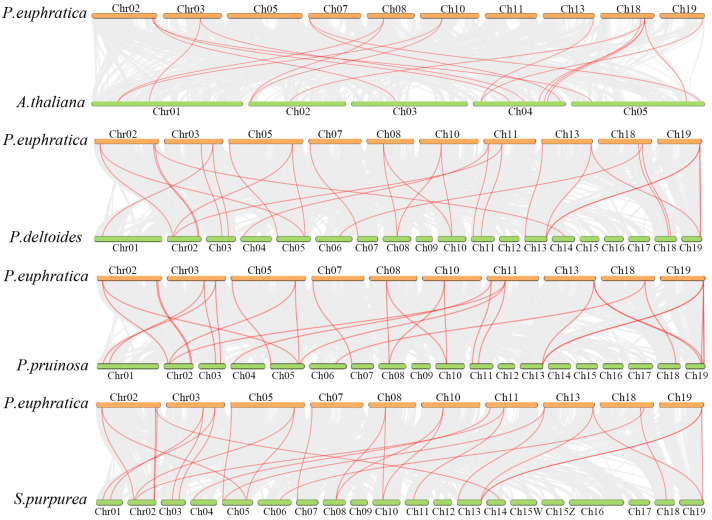
*XTH* colinear analysis among multiple species. Colinear analysis of *P. euphratica* and four other species, *A. thaliana*, *P. deltoides*, *P. pruinosa*, and *S. sinopurpurea*. Gray lines indicate colinear segments between the *P. euphratica* genome and those of other species, while red lines indicate *XTH* colinear pairs among species.

We performed interspecific collinearity analysis between *P. euphratica* and *A. thaliana*, as well as with *Populus deltoides* (a drought-tolerant poplar species of Salicaceae), *Populus pruinosa* (a drought-sensitive poplar species), and *Salix sinopurpurea* (a drought-sensitive willow species of Salicaceae). The analysis revealed 18 collinear gene pairs between *P. euphratica* and *A. thaliana* (Figure 4), whereas the numbers of collinear pairs between *P. euphratica* and *P. deltoides*, *P. pruinosa*, and *S. sinopurpurea* were 30, 28, and 29, respectively. These results indicate that the *XTH* gene family exhibits significantly higher evolutionary conservation within the Salicaceae family than across families (as represented by *A. thaliana*), suggesting that this gene family may have maintained greater structural stability through gene duplication and functional differentiation during adaptive evolution in woody plants.

### 2.5. Chromosomal Localization Analysis of PeXTHs

**Figure 5:**
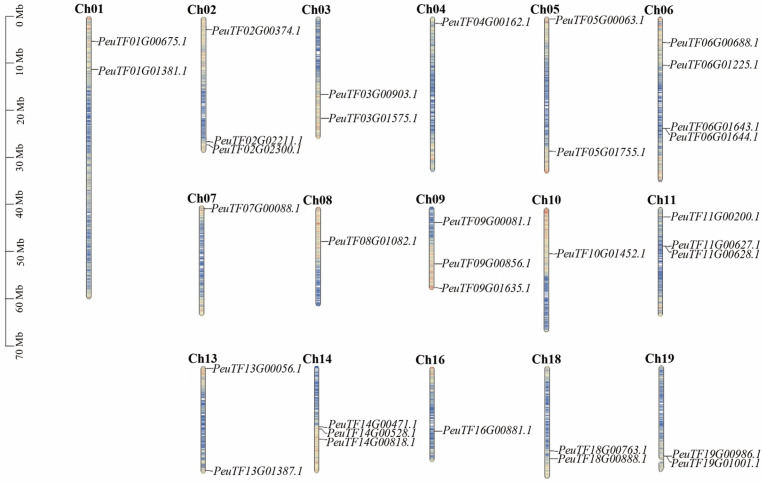
Chromosome location. Blue indicates low gene density on the chromosome, while red indicates high gene density on the chromosome.

Chromosomal localization analysis revealed that the 33 identified *PeXTH* genes are unevenly distributed across 16 chromosomes in *P*. *euphratica* (Figure 5). No family members were detected on chromosomes 12, 15, and 17. Gene distribution density varied among chromosomes, with chromosome 6 harboring the highest number (four *PeXTH* genes). Notably, two genes each were densely clustered on chromosomes 6, 11, and 19, suggesting potential hotspots for *XTH* gene localization in the *P. euphratica* genome. This genomic arrangement suggests that the proteins encoded by these genes may share functional similarities, reflecting potential evolutionary conservation within this gene family.

### 2.6. Phylogenetic Tree of PeXTHs

**Figure 6:**
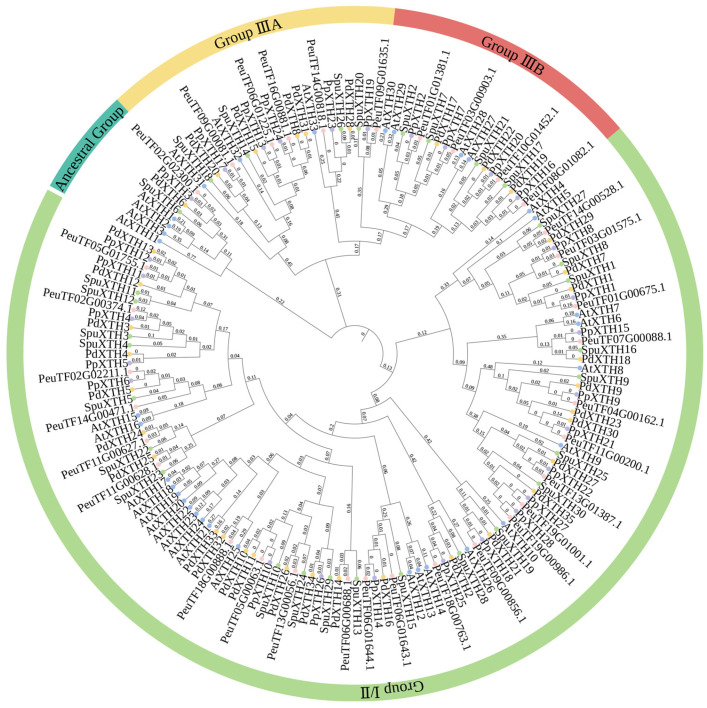
Phylogenetic tree of five species of *XTH* gene family members constructed using the neighbor-joining method. Phylogenetic analysis of XTH family protein sequences from five species (*P. euphratica*, *P. deltoides*, *P. pruinosa*, *S. sinopurpurea*, and *A. thaliana*) was performed using the ML (Maximum Likelihood) method by MEGA11 software (version 11.0.13). The value at the branch node represents the bootstrap value (calculated from 1000 repetitions) and shows only values greater than or equal to 50%. The number on the branch represents the branch confidence. Different colored circles represent different species. Species are color-coded as *P. deltoides* (orange), *A. thaliana* (blue), *S. sinopurpurea* (green), *P. pruinosa* (purple), and *P. euphratica* (pink); based on the grouping of *A. thaliana*, XTH was divided into four categories. Each group is distinguished by a different color.

A phylogenetic tree was constructed using the ML (Maximum Likelihood) method based on protein sequences of 33 *XTH* genes from *P*. *euphratica* and homologous sequences from other species (Figure 6). Following the classification scheme established for *AtXTHs*, the 159 *XTHs* members from five species were categorized into four distinct groups: Group I/II, Group IIIA, Group IIIB, and the Ancestral Group. Members of Groups I, II, and IIIB exhibit xyloglucan endotransglucosylase (XET) activity, enabling cleavage and religation of xyloglucan chains, whereas Group IIIA members display xyloglucan endohydrolase (XEH) activity, specifically hydrolyzing β-1,4-glycosidic bonds within xyloglucan polymers. Among the *PeXTHs*, 23, 5, 4, and 1 members were assigned to these subgroups, respectively. Given that AtXTHs within the same subgroup often share functional similarities, it is plausible that PeXTHs exhibit comparable functional conservation. Notably, XTHs from *P. euphratica* and *P. deltoides* formed highly supported clades, reflecting pronounced sequence homology and evolutionary conservation of function between these two species.

### 2.7. PeXTH Transcriptome Sequencing and Data Analysis

**Figure 7:**
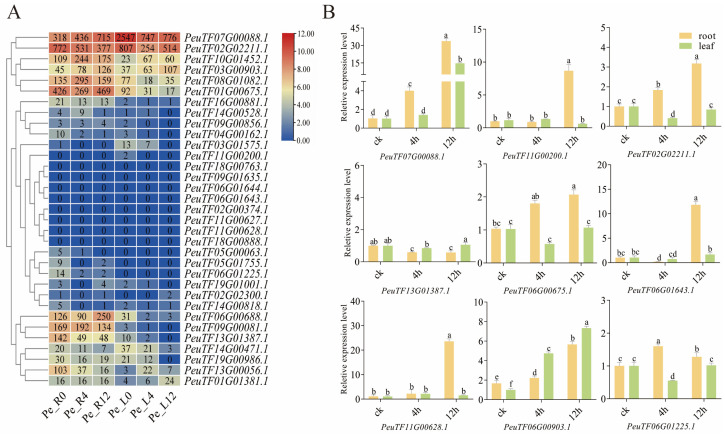
The expression pattern of *PeXTHs* under drought stress treatment. (**A**) Heat map of *PeXTHs* gene family member expression levels in *P. euphratica* seedlings under drought stress. Colors represent relative gene expression levels; red indicates high expression and blue indicates low expression. The number in the heat map is the original FPKM value. Pe-R0 represents gene expression levels in roots without drought treatment, and Pe-L0 represents expression levels in leaves without drought treatment. Pe-R4 indicates gene expression levels in roots after 4 h of drought treatment, while Pe-L4 indicates expression levels in leaves under the same conditions. Pe-R12 indicates gene expression levels in roots after 12 h of drought treatment, and Pe-L12 indicates expression levels in leaves under the same conditions. The color scale directly corresponds to the log_2_ (x + 1) converted value of FPKM. (**B**) Validation of the transcriptomic data by RT-qPCR is presented. Yellow and green columns represent the gene expression levels in the root and leaf, respectively. Letters (a, b, c, d) mark statistically distinct groups based on Duncan’s multiple range test.

To investigate the response of *PeXTH* genes to drought stress, we analyzed their transcript levels in *P*. *euphratica* seedlings under the drought treatment at 0, 4, and 12 h (Figure 7A). Visualization of the expression data using TBtools revealed that *PeuTF07G00088.1* and *PeuTF02G02211.1* exhibited the highest transcript abundance among all *PeXTH* genes. Notably, the expression level of *PeuTF07G00088.1* increased in roots but decreased in leaves under drought stress. In contrast, *PeuTF02G02211.1* showed declining expression trends in both roots and leaves. These results suggest that *PeuTF07G00088.1* may contribute to drought adaptation by enhancing its transcriptional activity in root tissues upon perception of drought signals.

To validate the transcriptome data, the expression patterns of nine *PeXTH* genes in *P*. *euphratica* seedlings under drought stress were analyzed using quantitative real-time PCR (RT-qPCR). The relative expression of 9 genes from different branches of the phylogenetic tree was measured under simulated drought stress of varying durations. After 12 h of treatment, most genes exhibited generally higher expression levels in roots than in leaves. Notably, *PeXTH7* (*PeuTF07G00088.1*) showed significant time-dependent upregulation in both roots and leaves following 4 h and 12 h of treatment with 25% PEG6000, reaching the highest expression level among all examined genes (Figure 7B). This expression trend was consistent with the transcriptome sequencing results. Furthermore, seven genes displayed significantly higher expression in roots compared to leaves at the end of the treatment period. Based on its markedly high expression in both roots and leaves, *PeXTH7* (*PeuTF07G00088.1*) was selected for further functional investigation.

### 2.8. Subcellular Localization of PeXTH7 in Nicotiana benthamiana

**Figure 8:**
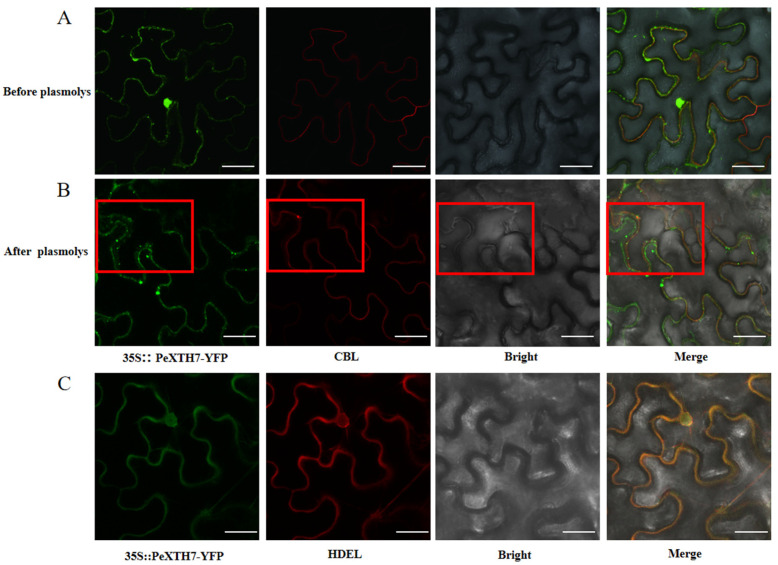
Subcellular localization of the 35S::*PeXTH7*-YFP protein in tobacco leaf epidermal cells. (**A**–**C**) Observation of membrane localization markers co-expressed with *PeXTH7* before and after plasmodesmal separation, as well as markers for the endoplasmic reticulum, and fusion images. (**A**) Before plasmodesmal separation, the cell membrane marker (CBL) overlaps with the YFP fluorescent label carried by *PeXTH7*. (**B**) After plasma membrane separation, the cell membrane marker (CBL) separates from the YFP fluorescent label carried by *PeXTH7*, The region enclosed by the red box exhibits distinct plasmolysis. (**C**) The endoplasmic reticulum marker (HDEL) fuses with the YFP fluorescent label carried by *PeXTH7* (Scale bar = 50 µm).

To further investigate the function of *PeXTH7* (*PeuTF07G00088.1*) and determine its subcellular distribution, we performed experimental validation of its localization. Agrobacterium tumefaciens GV3101 harboring the 35S::*PeXTH7*-YFP construct was infiltrated into *N*. *benthamiana* leaves, and the subcellular localization was examined using confocal laser scanning microscopy (Figure 8). The fluorescence signal of 35S::*PeXTH7*-YFP showed colocalization with both the plasma membrane and endoplasmic reticulum markers. However, following plasmolysis, the 35S::*PeXTH7*-YFP signal dissociated from the plasma membrane marker. These results indicate that *PeXTH7* is synthesized in the endoplasmic reticulum and subsequently functions in the cell wall.

### 2.9. Effect of PeXTH7 Overexpression on the Regulation of Root System Development in Transgenic A. thaliana

**Figure 9:**
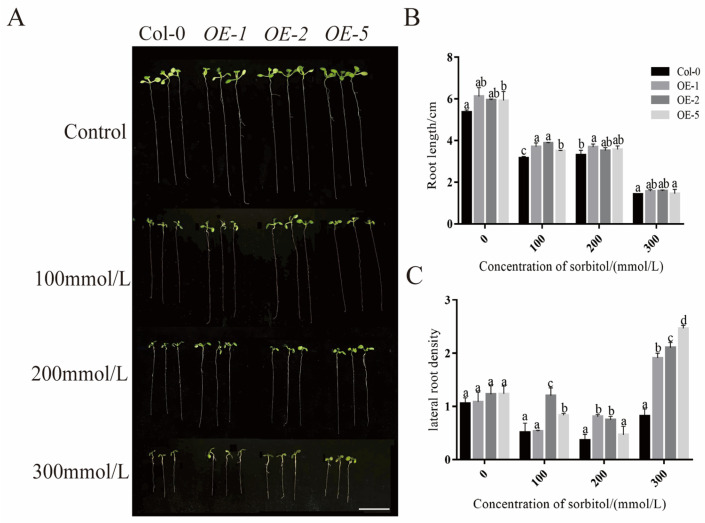
Overexpression of *PeXTH7* enhances root system development under simulated drought stress in *Arabidopsis*. (**A**) Representative root phenotypes of wild-type (Col-0) and three independent *PeXTH7*-overexpressing (OE-1, OE-2 and OE-5) lines under sorbitol-induced osmotic stress. Scale bar = 1 cm. (**B**) Primary root length of the plants shown in (**A**). (**C**) Primary lateral root density of the plants shown in (**A**). Data are presented as mean ± SEM (n ≥ 10). Bars marked with different letters are significantly different (*p* < 0.05, one-way ANOVA).

To determine the function of *PeXTH7* in root development, we generated transgenic *A. thaliana* (Col-0) plants overexpressing *PeXTH7* driven by the 35S promoter. These plants showed a significant increase in root length. Without sorbitol stress, the overexpression lines exhibited significantly longer primary roots along with an increased density of lateral roots, compared to Col-0 (Figure 9). Under sorbitol-induced osmotic stress, although root growth was suppressed across all lines, the *PeXTH7*-overexpressing lines consistently developed longer roots and sustained better root growth than Col-0. Statistical analysis of three randomly selected independent lines revealed significantly greater root lengths compared to the wild-type. Our results demonstrate that *PeXTH7* promotes root growth and development, which contributes to enhanced drought tolerance.

## 3. Discussion

### 3.1. Identification of 33 XTH Family Members in P. euphratica

*P*. *euphratica* is predominantly distributed in the Tarim River Basin of Xinjiang, China, where it plays a vital role in maintaining the structure and function of local ecosystems. Through its growth characteristics, it contributes to the regulation of river water resources and serves as an important ecological barrier. Exhibiting remarkable adaptability to the extreme desert conditions of northwestern China, *P. euphratica* has become a key model species for investigating the molecular and physiological mechanisms of abiotic stress tolerance in woody plants. It holds substantial value for research in related scientific fields.

Plant cell walls represent a unique extracellular matrix primarily composed of cellulose, hemicellulose, and pectin, typically supplemented with structural glycoproteins and lignin [29]. Xyloglucan is the main hemicellulose component of primary cell walls in plants [30]. This highly complex organization renders it a critical player in environmental stress perception. Multiple mechanisms regulate plant drought tolerance, with research by Hoson T demonstrating that the cell wall and plasma membrane collaboratively perceive external stress signals. Upon sensing environmental stresses, the mechanical properties of the cell wall undergo substantial modifications to mitigate adverse conditions [31].

Plant cell wall proteins (CWPs) play a major role in cell wall remodeling and signal transduction. Cell wall proteomics and numerous genetic and biochemical studies have revealed the high diversity of CWPs [32]. Enzymatically active protein components, including glycosidases, proteases, and transferases, occupy a central position in dynamic cell wall restructuring and environmental responses [33,34,35]; these enzymes regulate the assembly and disassembly of cell wall polysaccharide networks through spatiotemporally precise expression patterns [36]. Furthermore, protein–protein and protein–polysaccharide interactions can alter the mechanical properties of the wall and promote its supramolecular assembly [37,38,39]. These findings reveal how plant cells synthesize cell wall polysaccharides, assemble them into a sturdy fiber network, and regulate cell wall expansion during cell growth [40]. Xyloglucan endotransglucosylase/hydrolase (XTH) mediates the cleavage and re-ligation of xyloglucan, a key network component, thereby regulating cell wall extensibility while maintaining its structural integrity. Comparative genomic analyses suggest a non-plant origin for the XTH family, tracing back to Alphaproteobacteria ExoKs—bacterial enzymes involved in biofilm loosening. The earliest XTHs likely evolved in freshwater algae related to Cosmarium through C-terminal extension of EG16-2 proteins [41]. This ancient evolutionary origin established the foundation for its diverse functional roles in land plants. Previous studies have demonstrated that xyloglucan endotransglucosylase/hydrolase (XTH) contributes to cell wall biosynthesis and is closely associated with plant growth, development, and stress resilience, participating in processes such as root elongation [42], leaf venation patterning [43], fruit ripening [44], and responses to biotic and abiotic stresses [45,46].

Research on PeXTH functions remains relatively limited. In this study, we systematically identified XTH family members in four Salicaceae species: 33 in *P. euphratica*, 28 in *P. deltoides*, 30 in *S. sinopurpurea*, and 35 in *P. pruinosa*. Comparative analysis revealed limited variation in gene copy number among these species. The expansion in *P. pruinosa* may be attributed to gene duplication events during evolution, whereas the contraction in *P. deltoides* and *S. sinopurpurea* likely reflects lineage-specific selection associated with environmental adaptation. Furthermore, bioinformatic analyses of *PeXTHs*, including sequence conservation, gene structure, and potential functions, provide a theoretical foundation for elucidating the functional mechanisms of this gene family.

### 3.2. Potential Biological Functions of the PeXTH Gene Family

The *XTH* gene family plays crucial roles in plant growth and environmental adaptation. Proteins in this family exhibit three characteristic structural domains: (1) the DEIDFEFLG motif, responsible for transferase activity, in which the first glutamic acid (E) residue serves as a binding site essential for catalysis, while the second E functions as a proton donor [47]; (2) the N-glycosylation motif NXT/S, containing serine (Ser) and threonine (Thr) residues adjacent to the catalytic site, which is critical for enzymatic activity; and (3) a C-terminal cysteine residue capable of forming disulfide bonds that significantly contribute to protein structural stability [48]. Studies have revealed that different XTH members exhibit distinct responses to various hormones. Evidence indicates that gibberellic acid (GA_3_) regulates cell wall elongation by modulating the expression and activity of cell wall-modifying enzymes [49,50]. GA3 treatment of *A. thaliana* induced upregulation of *AtXTH21* expression in *A. thaliana* [51]. The expression levels of the rose genes *RbXTH1* and *RbXTH2* were upregulated after ethylene treatment [52]. The expression level of *A. thaliana AtXTH23* was significantly upregulated after treatment with abscisic acid (ABA) [15]. These findings collectively elucidate the molecular mechanisms through which the XTH family participates in plant growth, development, and environmental adaptation via specialized hormone regulatory networks.

Cis-acting elements represent specific DNA sequences situated in the 5′ upstream regulatory regions of structural genes, capable of binding transcription factors to modulate gene expression [53]. Analysis of the promoters of the *PeXTH* gene family revealed that most members contain light-responsive elements (e.g., G-box) and phytohormone-responsive elements (e.g., ABRE and CGTCA-motif), suggesting that their expression is likely regulated by light signaling, abscisic acid (ABA), and methyl jasmonate (MeJA). This inference is supported by previous studies in cherry (*Prunus avium* L.), where exogenous application of ABA, MeJA, and ethylene significantly upregulated the expression of *PavXTH15* [54,55]. Further examination identified that *PeXTH* promoters also harbor multiple cis-elements associated with abiotic stress responses, including drought-inducible elements (MBS), low-temperature response elements (LTR), and defense stress elements (TC-rich repeats), implying their broad involvement in plant adaptation to environmental challenges. Notably, the *PeXTH7* promoter is particularly enriched with stress-responsive elements: the MBS element mediates drought stress responses, while the ABRE element participates in the ABA signaling pathway, which plays a pivotal role in plant adaptation to drought and salt stress [56]. This ABRE-centered regulatory pattern shows remarkable consistency with the *XTH* gene *PagXTH12* in 84K poplar (*Populus alba* × *P. glandulosa*) [21], whose promoter also contains ABRE elements and exhibits significant upregulation under drought and salt stress conditions. Moreover, the presence of numerous growth- and development-related regulatory elements further underscores the functional complexity and diversity of this gene family.

A phylogenetic tree constructed using the neighbor-joining method, integrated with interspecies collinearity analysis, revealed high sequence homology and evolutionary conservation of the *XTH* gene family among closely related species. Phylogenetic classification indicated that *Arabidopsis thaliana XTH* members were distributed across the Group I/II, IIIA, IIIB, and Ancestral branches with 22, 3, 4, and 4 members, respectively, whereas *Populus euphratica* contained 23, 5, 4, and 1 members in the corresponding branches. Although the total number of *XTH* genes was similar between the two species, notable branch-specific distribution differences were observed: *P. euphratica* exhibited expansion in Groups I/II and IIIA but contraction in the Ancestral branch. This branch-specific expansion and contraction may have resulted from gene duplication or loss events during species differentiation [57]. Despite these interspecific distribution variations, analysis of protein characteristics demonstrated that although XTH proteins exhibit considerable polymorphism in molecular weight, isoelectric point, and amino acid sequence length, members within the same evolutionary branch share highly conserved protein motifs and gene structures, such as exon–intron organization, suggesting functional similarities among clade members. Conserved domain analysis further supported this functional differentiation pattern: 80% of Group IIIA members contain the LamG superfamily domain, whereas nearly all members of Groups I/II and IIIB harbor the GH16_XET functional domain. Gene duplication analysis revealed that the expansion of the *PeXTH* gene family primarily arose from segmental duplication events. All duplicated *PeXTH* gene pairs showed Ka/Ks ratios of less than 1.00 (Table 2), indicating that this gene family has undergone strong purifying selection during evolution. This selective pressure maintained relatively slow evolutionary rates and promoted functional conservation. Collectively, these findings demonstrate that the key functional domains and overall architecture of XTH proteins are highly conserved in *P*. *euphratica*, with purifying selection playing a critical evolutionary role in maintaining this conservation by effectively eliminating random amino acid mutations that could be detrimental to plant adaptability.

Previous studies have collectively demonstrated that *XTH* family members exhibit spatiotemporal expression specificity and participate in multiple plant growth and developmental processes, while being regulated by both hormonal and environmental cues [58]. Overexpression of the *CaXTH3* gene in *Arabidopsis thaliana* and chili peppers significantly enhanced transgenic plant tolerance to water loss, accompanied by increased mesophyll cell density and altered leaf morphology [24]. Earlier research also revealed that heterologous expression of a specific *P. euphratica XTH* gene in tobacco improved drought tolerance by reducing stomatal aperture and consequently decreasing water evaporation [59]. Additionally, studies have shown that *A*. *thaliana Atxth7* mutants exhibit shorter root length than wild-type plants under salt stress [60]. Based on the phylogenetic analysis and RT-qPCR results presented above, *PeuTF07G00088.1* (named *PeXTH7*) was ultimately selected from *Populus euphratica* for further investigation due to its highest homology with *AtXTH7*. Under drought stress conditions, *PeXTH7* showed sustained upregulation in both roots and leaves, with root tissues displaying significantly higher expression levels than leaf tissues. This tissue-specific expression pattern implies that *PeXTH7* may contribute to drought adaptation in *P. euphratica* primarily through regulating root architecture. Consequently, *PeXTH7* has been identified as a principal target for subsequent functional studies aimed at clarifying its precise role in drought resistance mechanisms of woody plants.

Plant growth and development fundamentally depend on cell wall expansion. Xyloglucan, a major hemicellulosic component of the cell wall, has long been a central focus of research. XTH enzymes play essential roles in xyloglucan cleavage and reconnection during cell wall remodeling. The plant XTH family comprises numerous members that are expressed across various tissues and organs, responding to hormonal and environmental stress signals, thereby underscoring their crucial functions in plant growth and adaptation. Although research on *XTH* genes has achieved considerable progress in recent years, the specific biological functions of this gene family remain incompletely characterized. In this study, we performed a genome-wide analysis of the *XTH* gene family in *P. euphratica*, identified all its members, and conducted preliminary predictions regarding the response of *PeXTH7* to drought stress. Future work should focus on elucidating the molecular mechanisms through which *PeXTH7* contributes to abiotic stress tolerance.

### 3.3. PeXTH7 Participates in the Regulation of Root Length in Transgenic A. thaliana

To assess whether *PeXTH7* shares a conserved role in root growth regulation, we measured root length and lateral root density in *PeXTH7*-overexpressing *A. thaliana* plants. The results revealed a significant increase in root length compared to wild-type controls. Given the close relationship between root length and water uptake efficiency, we propose that *PeXTH7* may enhance drought tolerance by modulating root elongation through cell wall regulation. However, the precise molecular mechanism underlying this process requires further investigation.

## 4. Materials and Methods

### 4.1. Genetic Identification, Physical and Chemical Property Analysis

The XTH protein sequences of *Arabidopsis thaliana* were retrieved from the TAIR database (https://www.arabidopsis.org/; accessed on 19 November 2024) and used to perform a website BLASTp search against the *Populus euphratica* protein database (https://ngdc.cncb.ac.cn/search/specific?db=bioproject&q=PRJCA006811; accessed on 19 November 2024) [13], with an E-value threshold set to 1 × 10^−5^. Subsequently, HMM profiles of the Glyco_hydro_16 (PF00722) and XET_C (PF06955) domains were obtained from the Pfam database (https://pfam.xfam.org/; accessed on 22 November 2024) for local HMM-based screening. The results from both approaches were combined, and sequences that did not simultaneously contain the Glyco_hydro_16 and XET_C domains were filtered out using the online bioinformatics tool NCBI CDD Search (https://www.ncbi.nlm.nih.gov/Structure/bwrpsb/bwrpsb.cgi; accessed on 22 November 2024). The remaining sequences were identified as putative *XTH* genes of *P. euphratica*. Based on genomic data available at the National Center for Biotechnology Information (NCBI), *XTH* family members were also identified in three additional poplar species: *P. deltoides*  (WV94_445), *P. pruinosa* (PRJNA863418), and *S. sinopurpurea* [61].

The physicochemical properties of the identified PeXTH proteins, including coding sequence length, amino acid number, molecular weight, and theoretical isoelectric point, were analyzed using the Expasy ProtParam tool (https://web.expasy.org/protparam/; accessed on 11 September 2024). Subcellular localization of *P. euphratica* XTH family members was predicted via WOLF PSORT (https://www.genscript.com/; accessed on 11 December 2024). Chromosomal location data were extracted from the genome annotation file, and the physical distribution of *XTH* genes was visualized using TBtools.

### 4.2. PeXTH Gene Structure and Conserved Motif Analysis

Conserved motifs in the *XTH* gene family proteins were analyzed using the MEME online tool (https://meme-suite.org/meme/tools/meme; accessed on 11 September 2024). The analysis was conducted with the optimum motif width set to 10–150 residues and the maximum number of motifs set to 10, while other parameters remained at their default values. The resulting gene structures and motif distributions were visualized using TBtools software (v2.119) for subsequent interpretation.

### 4.3. Analysis of Cis-Acting Elements of PeXTH Promoter

The promoter sequences of *PeXTH* genes, defined as the 2000 bp regions upstream of the translation start sites, were extracted using TBtools (v2.119). Putative cis-regulatory elements within these promoter sequences were identified using the PlantCARE database (https://bioinformatics.psb.ugent.be/webtools/plantcare/html; accessed on 18 October 2024). The predicted cis-acting elements were subsequently visualized using TBtools software (v2.119).

### 4.4. Collinearity and Chromosome Mapping Among Multiple Species

Orthologous gene pairs between *P. euphratica* and the other four species (*P. deltoides*, *P. pruinosa*, *S. sinopurpurea*, and *A. thaliana*) were identified using BLASTP alignment. Subsequently, TBtools (version 2.119) software was used to screen for collinearity blocks between *P. euphratica* and these four species and visualize them.

Chromosome length information (Fasta statistics), *PeXTH* gene IDs and positional data (GFF3 parsing/text block extraction and filtering), and gene density distribution were extracted from the *P. euphratica* genome using TBtools (v2.119). Subsequently, chromosomal localization patterns were visualized with the TBtools Gene Position Visualization function, and regional gene distributions were further analyzed using the same module.

### 4.5. PeXTH Phylogenetic Tree Analysis

Protein sequence clustering and phylogenetic analysis of *XTH* gene family members from *P. euphratica*, *P. pruinosa*, *P. deltoides*, *S. sinopurpurea*, and *A. thaliana* were performed using MEGA11 software (version 11.0.13). The phylogenetic tree was edited and beautified using the iTOL online tool (https://itol.embl.de/, accessed on 19 January 2025).

### 4.6. Transcriptome Sequencing and Data Analysis of PeXTHs

Transcriptome sequencing data were derived from four-month-old *P.euphratica* seedlings treated with 25% PEG 6000 and their control conditions. The expression profiles of *PeXTHs* gene family members were extracted from the quantitative expression data of each treatment [62]. *P. euphratica* seedlings were treated with a 25% polyethylene glycol 6000 solution, and leaf and root samples were collected at 0, 4, and 12 h post-treatment. The samples were immediately frozen in liquid nitrogen for storage and sequenced using the Ion Proton sequencing platform at the Shenzhen Huada Gene Research Institute. Gene expression levels were calculated using the FPKM (read per million mapped reads per thousand base pairs) method. The RNA-seq data have been uploaded to the NCBI database (https://www.ncbi.nlm.nih.gov/bioproject/PRJNA580347/, accessed on 11 September 2024). Transcriptome data visualization analysis was performed using TBtools software (version 2.119), and the data were subjected to numerical standardization and log transformation. The log transformation parameters were set as follows: base = 2.0; offset = 1.0.

*P*. *euphratica* seedlings were grown under a 16-h photoperiod at 25 ± 1 °C and 75% relative humidity, with bi-weekly irrigation. After three months, seedlings were treated with 25% PEG6000 for 0, 4, and 12 h (n = 3 per group). Post-treatment, roots and leaves were sampled, flash-frozen in liquid nitrogen, and stored at −80 °C. Total RNA was extracted using a SPARKscript II RT Plus Kit (SparkJade, Jinan, China), and cDNA was synthesized from 1 µg RNA. RT-qPCR was carried out in 10 µL reactions with SYBR Green mix and gene-specific primers (Table S1). Amplification was achieved using the following cycle settings: 95 °C for 30 s, then 45 cycles of 95 °C for 10 s, 55 °C for 10 s, and 72 °C for 30 s for plate reading. RT-qPCR yielded products between 80 bp and 150 bp in size. After normalization with *PeActin*, relative expression was assessed. The 2^−ΔΔCt^ method was applied to data evaluation.

### 4.7. Subcellular Localization of PeXTH7

The constructed 35S::*PeXTH7*-YFP vector was transformed into *Agrobacterium tumefaciens* strain GV3101. Strains carrying the target plasmid (CBL-mCherry) were cultured overnight in LB medium supplemented with appropriate antibiotics. Healthy leaves from 4-week-old tobacco plants were selected for infiltration. Agrobacterium cultures containing *PeXTH7* and the helper strain 19K (used to enhance fluorescence signals) were grown overnight in LB liquid medium. When the OD_600_ of the PeXTH7-containing Agrobacterium culture reached 1.0–1.2, cells were harvested by centrifugation at 10,000 rpm for 1–2 min.

The supernatant was discarded and the bacterial cells were collected. An infiltration buffer containing MES, MgCl_2_·6H_2_O, and acetosyringone was prepared and adjusted to pH 6.0 using KOH. This buffer was mixed with the *Agrobacterium* suspension and incubated at 28 °C for 1 h. The bacterial suspension was then infiltrated into the abaxial surface of tobacco leaves, with injection sites clearly marked. Following 24 h of dark treatment, the plants were transferred to a controlled-environment growth chamber for 2–3 days. Subcellular localization was analyzed using a laser scanning confocal microscope (Nikon TS100, Tokyo, Japan). For YFP signal detection, an excitation wavelength of 514 nm was used with emission collection between 524–574 nm. After inducing plasmolysis with 30% sucrose solution, fluorescence distribution was re-evaluated under the same optical settings. For visualization of the endoplasmic reticulum marker, excitation at 587 nm was employed with emission detection in the 597–650 nm range.

### 4.8. Effect of PeXTH7 Overexpression on Root Length in A. thaliana

The wild-type A. thaliana seeds used in this study was the Columbia-0 (Col-0) ecotype. The *PeXTH7* gene was heterologously expressed in Arabidopsis using the pgreenII 0179 (35S NOS)-YFP overexpression vector in the GV3101 Agrobacterium tumefaciens strain, with *Escherichia coli*—TOP10 used for plasmid propagation. The detailed transgenic methodology followed a previously described protocol [63]. Seeds of the generated lines—Col-0 and three independent *PeXTH7*-overexpressing lines (OE-1, OE-2, OE-5)—were surface-sterilized and sown on 1/2 MS medium. For each line, 20 seeds were plated per biological replicate, with five replicates established per treatment. A 0 mmol/L sorbitol condition served as the control. After stratification at 4 °C for 2–3 days, the plates were transferred to a growth chamber and positioned vertically. Following 4 days of growth under standard conditions, healthy and uniformly sized seedlings were selected and carefully transferred to fresh 1/2 MS media containing either 0, 100, 200, or 300 mmol/L sorbitol. The seedlings were grown for an additional 5 days, after which representative healthy seedlings from each group were photographed. Primary root length was subsequently measured and analyzed from the images using the software ImageJ (version 1.44p).

## 5. Conclusions

In this study, a total of 33 *PeXTHs* genes were identified in *P. euphratica*, and their gene structures, phylogenetic relationships, collinearity, and cis-acting elements were systematically analyzed. The results indicated that *PeXTHs* gene family members are involved in drought resistance by regulating root growth and development. RNA sequencing and RT-qPCR analyses further confirmed the critical role of *PeXTH7* in the drought stress response. Heterologous overexpression of *PeXTH7* in wild-type Arabidopsis significantly promoted root elongation and enhanced drought tolerance. These findings suggest that *PeXTH7* may improve drought adaptability in *P. euphratica* by facilitating root system development. Therefore, this study not only provides a theoretical basis for understanding the function of *PeXTHs* genes in root growth, but also provides a reference for the subsequent exploration of the molecular response of *P. euphratica* to abiotic stress.

## Figures and Tables

**Table 1:** Physical and chemical properties of the *PeXTH* gene family.

Gene ID	Molecular Weight (kDa)	Number of Amino Acid (aa)	Aliphatic Index	Theoretical pI	Grand Average of Hydropathicity	Predicted Location (s)
PeuTF09G00081.1	33.05	294	68.03	6.15	−0.486	Cell wall
PeuTF18G00763.1	33.19	291	62.71	8.79	−0.411	Cell wall
PeuTF07G00088.1	24.7	213	60.05	5.95	−0.546	Cell wall
PeuTF09G01635.1	32.28	289	62.77	5.03	−0.362	Cell wall
PeuTF06G01644.1	32.66	287	69.62	6.59	−0.307	Cell wall, Cytoplasm
PeuTF06G01643.1	32.76	287	68.95	7.04	−0.356	Cell wall, Cytoplasm
PeuTF09G00856.1	34.1	294	65.31	8.9	−0.494	Cell wall
PeuTF08G01082.1	38	336	71.37	5.88	−0.346	Cell wall
PeuTF01G00675.1	33.55	289	59.38	6.22	−0.512	Cell wall, Cytoplasm
PeuTF13G00056.1	23.83	210	55.81	5.71	−0.561	Cell wall, Cytoplasm
PeuTF11G00200.1	34.94	299	56.45	5.09	−0.585	Cell wall
PeuTF04G00162.1	34.1	293	56.62	4.76	−0.561	Cell wall
PeuTF02G00374.1	25.69	224	83.57	8.51	−0.242	Cell wall
PeuTF05G01755.1	33.24	295	70.75	8.84	−0.322	Cell wall, Cytoplasm
PeuTF10G01452.1	38.21	335	71.28	6.07	−0.417	Cell wall
PeuTF01G01381.1	39.49	338	57.43	9.1	−0.559	Cell wall
PeuTF11G00627.1	33.37	292	71.44	8.99	−0.399	Cell wall
PeuTF11G00628.1	31.54	274	75.44	9.67	−0.419	Cell wall
PeuTF03G01575.1	35	300	65.33	8.4	−0.387	Cell wall, Cytoplasm
PeuTF14G00471.1	32.25	282	66.77	9.07	−0.33	Cell wall, Cytoplasm
PeuTF14G00528.1	30.82	264	67.95	8.87	−0.539	Cell wall, Cytoplasm
PeuTF03G00903.1	36.3	311	62.77	9	−0.603	Cell wall
PeuTF06G01225.1	33.88	294	60.75	9.58	−0.443	Cell wall
PeuTF02G02211.1	33.03	288	66.04	9.24	−0.34	Cell wall, Cytoplasm
PeuTF02G02300.1	33.55	293	58.6	5.64	−0.606	Cell wall
PeuTF06G00688.1	31.88	285	62.67	7.58	−0.322	Cell wall, Cytoplasm
PeuTF14G00818.1	34.88	311	70.84	6.49	−0.251	Cell wall
PeuTF19G00986.1	33.78	296	69.8	5.89	−0.295	Cell wall
PeuTF13G01387.1	28.12	246	58.66	5.8	−0.498	Cell wall
PeuTF16G00881.1	34.03	294	61.7	9.39	−0.426	Cell wall
PeuTF18G00888.1	33.23	292	71.16	8.95	−0.383	Cell wall, Cytoplasm
PeuTF05G00063.1	33.01	291	71.41	4.48	−0.261	Cell wall, Cytoplasm
PeuTF19G01001.1	33.78	296	69.8	5.89	−0.295	Cell wall

**Table 2:** Evolutionary selection pressures of XTHs in *P*. *euphratica*.

Gene_1	Gene_2	Ka	Ks	Ka/Ks
PeuTF01G01381.1	PeuTF03G00903.1	0.088029963	0.336819885	0.261356192
PeuTF01G00675.1	PeuTF03G01575.1	0.136741168	0.46409146	0.294642715
PeuTF08G01082.1	PeuTF10G01452.1	0.052476004	0.350912053	0.149541755
PeuTF06G01643.1	PeuTF18G00888.1	0.240293693	2.165085817	0.110985759
PeuTF06G01225.1	PeuTF09G00081.1	0.197261823	1.201835773	0.164133759
PeuTF06G01225.1	PeuTF16G00881.1	0.038836235	0.416044352	0.093346383
PeuTF19G01001.1	PeuTF13G01387.1	0.060220985	0.446202234	0.134963432
PeuTF14G00528.1	PeuTF02G02300.1	0.427698912	NaN	NaN
PeuTF05G01755.1	PeuTF11G00627.1	0.211307847	NaN	NaN
PeuTF05G01755.1	PeuTF02G00374.1	0.109048588	0.500680619	0.217800697
PeuTF05G00063.1	PeuTF13G00056.1	0.126769113	0.411406195	0.30813613
PeuTF02G00374.1	PeuTF11G00627.1	0.233638517	NaN	NaN
PeuTF04G00162.1	PeuTF11G00200.1	0.038976214	0.365085239	0.106759218

Non-synonymous is abbreviated as Ka and synonymous is abbreviated as Ks.

## Data Availability

No large datasets were created in this study.

## Supplementary Materials

The following supporting information can be downloaded at https://www.mdpi.com/article/10.3390/plants14243847/s1, Table S1: Primer Sequences for RT-qPCR.

## References

[1] Fang Y., Xiong L. (2015). General mechanisms of drought response and their application in drought resistance improvement in plants. Cell. Mol. Life Sci..

[2] Muhammad Aslam M., Waseem M. (2022). Mechanisms of Abscisic Acid-Mediated Drought Stress Responses in Plants. Int. J. Mol. Sci..

[3] Taylor-Teeples M., Lin L., de Lucas M., Turco G., Toal T.W., Gaudinier A., Young N.F., Trabucco G.M., Veling M.T., Lamothe R. (2015). An *Arabidopsis* gene regulatory network for secondary cell wall synthesis. Nature.

[4] Zhao C., Zayed O., Yu Z., Jiang W., Zhu P., Hsu C.C., Zhang L., Tao W.A., Lozano-Durán R., Zhu J.K. (2018). Leucine-rich repeat extensin proteins regulate plant salt tolerance in *Arabidopsis*. Proc. Natl. Acad. Sci. USA.

[5] Sivan P., Urbancsok J., Donev E.N., Derba-Maceluch M., Barbut F.R. (2025). Modification of xylan in secondary walls alters cell wall biosynthesis and wood formation programs and improves saccharification. Plant Biotechnol. J..

[6] Feng W., Lindner H. (2016). Growing Out of Stress: The Role of Cell- and Organ-Scale Growth Control in Plant Water-Stress Responses. Plant Cell Physiol..

[7] De Caroli M., Manno E., Piro G. (2021). Ride to cell wall: *Arabidopsis* XTH11, XTH29 and XTH33 exhibit different secretion pathways and responses to heat and drought stress. Plant J. Cell Mol. Biol..

[8] Nishitani K., Tominaga R. (1992). Endo-xyloglucan transferase, a novel class of glycosyltransferase that catalyzes transfer of a segment of xyloglucan molecule to another xyloglucan molecule. J. Biol. Chem..

[9] Zhai J., Li Z., Si J., Zhang S., Han X., Chen X. (2022). Structural and Functional Responses of the Heteromorphic Leaves of Different Tree Heights on *Populus euphratica* Oliv. to Different Soil Moisture Conditions. Plants.

[10] Ge X.L., Zhang L., Du J.J., Wen S.S., Qu G.Z., Hu J.J. (2022). Transcriptome Analysis of *Populus euphratica* under Salt Treatment and PeERF1 Gene Enhances Salt Tolerance in Transgenic *Populus alba* × *Populus glandulosa*. Int. J. Mol. Sci..

[11] Korver R.A., Koevoets I.T., Testerink C. (2018). Out of Shape During Stress: A Key Role for Auxin. Trends Plant Sci..

[12] Cao D., Li J., Huang Z., Baskin C.C., Baskin J.M., Hao P., Zhou W., Li J. (2012). Reproductive characteristics of a *Populus euphratica* population and prospects for its restoration in China. PLoS ONE.

[13] Zhang Z., Chen Y., Zhang J., Ma X., Li Y., Li M., Wang D., Kang M., Wu H., Yang Y. (2020). Improved genome assembly provides new insights into genome evolution in a desert poplar (*Populus euphratica*). Plant Physiol..

[14] Lin Z., Guo Y., Zhang R., Li Y., Wu Y., Sheen J. (2024). ABA-activated low-nanomolar Ca^2+^-CPK signalling controls root cap cycle plasticity and stress adaptation. Nat. Plants.

[15] Yokoyama R., Nishitani K. (2001). A comprehensive expression analysis of all members of a gene family encoding cell-wall enzymes allowed us to predict cis-regulatory regions involved in cell-wall construction in specific organs of *Arabidopsis*. Plant Cell Physiol..

[16] Yokoyama R., Rose J.K., Nishitani K. (2004). A Surprising Diversity and Abundance of Xyloglucan Endotransglucosylase/Hydrolases in Rice. Classification and Expression Analysis. Plant Physiol..

[17] Yang L., Chen Y., Liu X., Zhang S., Han Q. (2023). Genome-wide identification and expression analysis of xyloglucan endotransglucosylase/hydrolase genes family in *Salicaceae* during grafting. BMC Genom..

[18] Liu Y., Liu D., Zhang H., Gao H., Guo X., Wang D., Zhang X., Zhang A. (2007). The alpha- and beta-expansin and xyloglucan endotransglucosylase/hydrolase gene families of wheat: Molecular cloning, gene expression, and EST data mining. Genomics.

[19] Schultink A., Liu L., Zhu L., Pauly M. (2014). Structural Diversity and Function of Xyloglucan Sidechain Substituents. Plants.

[20] Yang Y., Miao Y., Zhong S., Fang Q., Wang Y., Dong B., Zhao H. (2022). Genome-Wide Identification and Expression Analysis of XTH Gene Family during Flower-Opening Stages in *Osmanthus fragrans*. Plants.

[21] Li Q., Li H., Yin C., Wang X., Jiang Q., Zhang R., Ge F., Chen Y., Yang L. (2019). Genome-Wide Identification and Characterization of Xyloglucan Endotransglycosylase/Hydrolase in *Ananas comosus* during Development. Genes.

[22] Yang Z., Zhang R., Zhou Z. (2022). The XTH Gene Family in *Schima superba*: Genome-Wide Identification, Expression Profiles, and Functional Interaction Network Analysis. Front. Plant Sci..

[23] Saladié M., Rose J.K., Cosgrove D.J., Catalá C. (2006). Characterization of a new xyloglucan endotransglucosylase/hydrolase (XTH) from ripening tomato fruit and implications for the diverse modes of enzymic action. Plant J. Cell Mol. Biol..

[24] Choi J.Y., Seo Y.S., Kim S.J., Kim W.T., Shin J.S. (2011). Constitutive expression of CaXTH3, a hot pepper xyloglucan endotransglucosylase/hydrolase, enhanced tolerance to salt and drought stresses without phenotypic defects in tomato plants (*Solanum lycopersicum* cv. Dotaerang). Plant Cell Rep..

[25] Chen J., Wan H., Zhao H., Dai X., Wu W., Liu J., Xu J., Yang R., Xu B., Zeng C. (2024). Identification and expression analysis of the Xyloglucan transglycosylase/hydrolase (XTH) gene family under abiotic stress in oilseed (*Brassica napus* L.). BMC Plant Biol..

[26] Zhang S., Wu Z. (2022). Chromosome-scale assemblies of the male and female *Populus euphratica* genomes reveal the molecular basis of sex determination and sexual dimorphism. Commun. Biol..

[27] Sun J., Xu J., Qiu C., Zhai J., Zhang S., Zhang X., Wu Z., Li Z. (2024). The chromosome-scale genome and population genomics reveal the adaptative evolution of *Populus pruinosa* to desertification environment. Hortic. Res..

[28] Wu Z., Jiang Z. (2023). Multi-omics analysis reveals spatiotemporal regulation and function of heteromorphic leaves in Populus. Plant Physiol..

[29] Zhang B., Gao Y., Zhang L., Zhou Y. (2021). The plant cell wall: Biosynthesis, construction, and functions. J. Integr. Plant Biol..

[30] Cosgrove D.J. (2024). Structure and growth of plant cell walls. Proc. Natl. Acad. Sci. USA.

[31] Hoson T. (1998). Apoplast as the site of response to environmental signals. J. Plant Res..

[32] Barbut F.R. (2022). Plant-Insect Interactions. Plants.

[33] Zhong R., Ye Z.H. (2015). Secondary cell walls: Biosynthesis, patterned deposition and transcriptional regulation. Plant Cell Physiol..

[34] Jamet E., Canut H., Boudart G., Pont-Lezica R.F. (2006). Cell wall proteins: A new insight through proteomics. Trends Plant Sci..

[35] Calderan-Rodrigues M.J., Guimarães Fonseca J., de Moraes F.E., Vaz Setem L., Carmanhanis Begossi A., Labate C.A. (2019). Plant Cell Wall Proteomics: A Focus on Monocot Species, *Brachypodium distachyon*, *Saccharum* spp. and *Oryza sativa*. Int. J. Mol. Sci..

[36] Franková L., Fry S.C. (2013). Biochemistry and physiological roles of enzymes that ‘cut and paste’ plant cell-wall polysaccharides. J. Exp. Bot..

[37] Hijazi M., Roujol D., Nguyen-Kim H., Del Rocio Cisneros Castillo L., Saland E., Jamet E., Albenne C. (2014). Arabinogalactan protein 31 (AGP31), a putative network-forming protein in *Arabidopsis thaliana* cell walls. Ann. Bot..

[38] Tan L., Eberhard S., Pattathil S., Warder C., Glushka J., Yuan C., Hao Z., Zhu X., Avci U., Miller J.S. (2013). An *Arabidopsis* cell wall proteoglycan consists of pectin and arabinoxylan covalently linked to an arabinogalactan protein. Plant Cell.

[39] Cannon M.C., Terneus K., Hall Q., Tan L., Wang Y., Wegenhart B.L., Chen L., Lamport D.T., Chen Y., Kieliszewski M.J. (2008). Self-assembly of the plant cell wall requires an extensin scaffold. Proc. Natl. Acad. Sci. USA.

[40] Cosgrove D.J. (2005). Growth of the plant cell wall. Nat. Rev. Mol. Cell Biol..

[41] Shinohara N., Nishitani K. (2021). Cryogenian Origin and Subsequent Diversification of the Plant Cell-Wall Enzyme XTH Family. Plant Cell Physiol..

[42] Gao Y., Wang L., Li D., Qi D., Fang F., Luo Y., Zhang H., Zhang S. (2024). Genome-wide characterization of the xyloglucan endotransglucosylase/hydrolase family genes and their response to plant hormone in sugar beet. Plant Physiol. Biochem..

[43] Sasidharan R., Keuskamp D.H., Kooke R., Voesenek L.A., Pierik R. (2014). Interactions between auxin, microtubules and XTHs mediate green shade- induced petiole elongation in *Arabidopsis*. PLoS ONE.

[44] Han Y., Han S., Ban Q., He Y., Jin M., Rao J. (2017). Overexpression of persimmon DkXTH1 enhanced tolerance to abiotic stress and delayed fruit softening in transgenic plants. Plant Cell Rep..

[45] Li Q., Hu A., Dou W., Qi J., Long Q., Zou X., Lei T., Yao L., He Y., Chen S. (2019). Systematic Analysis and Functional Validation of Citrus XTH Genes Reveal the Role of Csxth04 in Citrus Bacterial Canker Resistance and Tolerance. Front. Plant Sci..

[46] Qiao T., Zhang L., Yu Y., Pang Y., Tang X., Wang X., Li L., Li B., Sun Q. (2022). Identification and expression analysis of xyloglucan endotransglucosylase/hydrolase (XTH) family in grapevine (*Vitis vinifera* L.). PeerJ.

[47] Geisler-Lee J., Geisler M., Coutinho P.M., Segerman B., Nishikubo N., Takahashi J., Aspeborg H., Djerbi S., Master E., Andersson-Gunnerås S. (2006). Poplar carbohydrate-active enzymes. Gene identification and expression analyses. Plant Physiol..

[48] Thompson J.E., Fry S.C. (2001). Restructuring of wall-bound xyloglucan by transglycosylation in living plant cells. Plant J. Cell Mol. Biol..

[49] Sauret-Güeto S., Calder G., Harberd N.P. (2012). Transient gibberellin application promotes *Arabidopsis thaliana* hypocotyl cell elongation without maintaining transverse orientation of microtubules on the outer tangential wall of epidermal cells. Plant J. Cell Mol. Biol..

[50] Genovesi V., Fornalé S., Fry S.C., Ruel K., Ferrer P., Encina A., Sonbol F.M., Bosch J., Puigdomènech P., Rigau J. (2008). ZmXTH1, a new xyloglucan endotransglucosylase/hydrolase in maize, affects cell wall structure and composition in *Arabidopsis thaliana*. J. Exp. Bot..

[51] Liu Y.B., Lu S.M., Zhang J.F., Liu S., Lu Y.T. (2007). A xyloglucan endotransglucosylase/hydrolase involves in growth of primary root and alters the deposition of cellulose in *Arabidopsis*. Planta.

[52] Singh A.P., Tripathi S.K., Nath P., Sane A.P. (2011). Petal abscission in rose is associated with the differential expression of two ethylene-responsive xyloglucan endotransglucosylase/hydrolase genes, RbXTH1 and RbXTH2. J. Exp. Bot..

[53] Wittkopp P.J., Kalay G. (2011). Cis-regulatory elements: Molecular mechanisms and evolutionary processes underlying divergence. Nat. Rev. Genet..

[54] Thessen A.E., Cooper L., Swetnam T.L., Hegde H., Reese J., Elser J., Jaiswal P. (2023). Using knowledge graphs to infer gene expression in plants. Front. Artif. Intell..

[55] Zhai Z., Feng C. (2021). Genome-Wide Identification of the Xyloglucan endotransglucosylase/Hydrolase (XTH) and Polygalacturonase (PG) Genes and Characterization of Their Role in Fruit Softening of Sweet Cherry. Int. J. Mol. Sci..

[56] Zhu J.K. (2016). Abiotic Stress Signaling and Responses in Plants. Cell.

[57] Zhai X., Wang X., Yang X., Huang Q., Wu D., Wang Y., Kang H., Sha L., Fan X., Zhou Y. (2024). Genome-wide identification of bHLH transcription factors and expression analysis under drought stress in *Pseudoroegneria libanotica* at germination. Physiol. Mol. Biol. Plants Int. J. Funct. Plant Biol..

[58] Yokoyama R., Nishitani K. (2001). Endoxyloglucan transferase is localized both in the cell plate and in the secretory pathway destined for the apoplast in tobacco cells. Plant Cell Physiol..

[59] Han Y., Wang W., Sun J., Ding M., Zhao R., Deng S., Wang F., Hu Y., Wang Y., Lu Y. (2013). *Populus euphratica* XTH overexpression enhances salinity tolerance by the development of leaf succulence in transgenic tobacco plants. J. Exp. Bot..

[60] Cai H., Xu Y., Yan K., Zhang S., Yang G., Wu C., Zheng C. (2023). BREVIPEDICELLUS Positively Regulates Salt-Stress Tolerance in *Arabidopsis thaliana*. Int. J. Mol. Sci..

[61] Zhou R., Zhou R., Macaya-Sanz D., Carlson C.H., Schmutz J. (2020). A willow sex chromosome reveals convergent evolution of complex palindromic repeats. Nat. Plants.

[62] Jiao P., Wu Z., Wang X., Jiang Z., Wang Y., Liu H., Qin R., Li Z. (2021). Short-term transcriptomic responses of *Populus euphratica* roots and leaves to drought stress. J. For. Res..

[63] Qu Y., Bi C., He B., Ye N., Yin T., Xu L.A. (2019). Genome-wide identification and characterization of the MADS-box gene family in Salix suchowensis. PeerJ.

