# Supplementary material for: Identification of the Populus euphratica XTHs Gene Family and the Response of PeXTH7 to Abiotic Stress
Source: Plants (Basel). 2025 Dec 17;14(24):3847. doi: 10.3390/plants14243847 (PMC12737017; doi:10.3390/plants14243847)
Supplement: Supplementary file 1 [file plants-14-03847-s001.zip › plants-3992297-supplementary.pdf]

Table S1: Primer Sequences for qRT-PCR

Primer Name	Primer Sequences
PeuTF07G00088.1-F	ACCGTGGAAATTGGTGGGAA
PeuTF07G00088.1-R	ACTCTGGTGGTGTAGTCGGA
PeuTF11G00200.1-F	GTGTACAGAGAATGGGGCAG
PeuTF11G00200.1-R	ATAGGTGTGGTACTCCTCGG
PeuTF02G02211.1-F	GCGACCCTTACATTTTGCAC
PeuTF02G02211.1-R	TTCTTGTGGGGTCAAACCAG
PeuTF13G01387.1-F	AGCGGCATGTTGTGTTTCTA
PeuTF13G01387.1-R	GAGCTGTATACGCCCATAGC
PeuTF06G00675.1-F	AAGGCGCCCTTTATAGCTTC
PeuTF06G00675.1-R	GCACAGAATTTCGCTTCCAC
PeuTF06G01643.1-F	AGTGGTCAGCCATACACTCT
PeuTF06G01643.1-R	CAGTGGGGTCAAACCAAAGA
PeuTF11G00628.1-F	AAGATACTACGCCGAGGGAA
PeuTF11G00628.1-R	GTTTCCAGGGACAAGCTTCA
PeuTF06G00903.1-F	GAAGAAATGGGCAGCGAGTA
PeuTF06G00903.1-R	GGAAATTCCTCGATGGGGTC
PeuTF06G01225.1-F	GACGATGTGCCCATAAGGAG
PeuTF06G01225.1-R	GTAAACCCACATTGGCCTGA
PeActin-F	GTCCTCTTCCAGCCATCTC
PeActin-R	TTCGGTCAGCAATACCAGG
